# Trends in measuring BMR and RMR after spinal cord injury: a comprehensive review

**DOI:** 10.1017/S0007114523000831

**Published:** 2023-11-28

**Authors:** Ahmad M. Alazzam, Malak W. Alrubaye, Jacob A. Goldsmith, Ashraf S. Gorgey

**Affiliations:** 1 Spinal Cord Injury and Disorders Center, Hunter Holmes McGuire VA Medical Center, Richmond, VA, USA; 2 Physical Medicine and Rehabilitation, Virginia Commonwealth University, Richmond, VA, USA

**Keywords:** Obesity, BMR, RMR, Spinal cord injury

## Abstract

Studying factors that contribute to our understanding of maintaining normal energy balance are of paramount significance following spinal cord injury (SCI). Accurate determination of energy needs is crucial for providing nutritional guidance and managing the increasing prevalence of malnutrition or obesity after SCI. BMR represents 75–80 % of the total energy expenditure in persons with SCI. Accurately measuring BMR is an important component for calculating total energetic needs in this population. Indirect calorimetry is considered the gold-standard technique for measuring BMR. However, technical challenges may limit its applications in large cohort studies and alternatively rely on prediction equations. Previous work has shown that BMR changes in response to disuse and exercise in the range of 15–120 %. Factors including sex, level of injury and type of assistive devices may influence BMR after SCI. RMR is erroneously used interchangeably for BMR, which may result in overestimation of energetic intake when developing nutritional plans. To address this concern, we comprehensively reviewed studies that conducted BMR (*n*=15) and RMR (*n*=22) in persons with SCI. The results indicated that RMR is 9 % greater than BMR in persons with SCI. Furthermore, the SCI-specific prediction equations that incorporated measures of fat-free mass appeared to accurately predict BMR. Overall, the current findings highlighted the significance of measuring BMR as well as encouraging the research and clinical community to effectively establish countermeasures to combat obesity after SCI.

Alterations in body composition and metabolism occur following spinal cord injury (SCI)^(1,2)^. Adiposity increases while lean mass (LM) below the level of injury decreases, resulting in neurogenic obesity^(2–5)^. This reduction in LM with concomitant decreases in activity levels and impaired sympathetic nervous system activity results in a lowering of BMR and daily energy needs following SCI^(6,7)^. Persons with SCI report total energetic intakes lower than able-bodied (AB) individuals^(1,7,8)^ in addition to lower measured BMR and calculated total energy expenditure^(9,10)^. In spite of this reduced energetic intake, obesity is more prevalent among persons with chronic SCI compared with AB persons^(4,5,11)^. Across studies, it has been found that the prevalence of obesity varies from 40 to 66 % among individuals with SCI, and consequently, obese individuals with SCI are susceptible to a wide range of health consequences^(12,13)^.

Persons with chronic SCI have an increased risk for obesity-related cardiometabolic diseases, including dyslipidemia, glucose intolerance and diabetes mellitus, central obesity, systemic inflammation and mitochondrial dysfunction^(13–17)^. Additionally, reduced mobility due to chronic SCI predisposes individuals to adipose tissue accumulation and further increases the risk for obesity-related chronic diseases^(9,18)^. To counteract this increased risk, it is recommended that those with SCI engage in regular physical activity and modify their dietary habits^(10)^. However, recommended nutritional intake after SCI requires an accurate measurement of metabolic rate and appropriate classification of SCI-specific obesity^(19)^. The purpose of this review is to summarise the current SCI literature pertaining to metabolic rate and systematically highlight those differences in measuring BMR compared with RMR in persons with SCI. Therefore, researchers and clinicians should rely on measured or predicted BMR when developing dietary regimens in order to promote weight loss in persons with SCI as RMR may increase the risk of overfeeding.

## Management of neurogenic obesity after spinal cord injury

The lack of innervation to paralysed limbs results in extensive muscle atrophy and progressive accumulation of FM and ectopic adiposity^(3)^; which in combination with a sedentary lifestyle is responsible for dysregulated carbohydrate, protein and fat metabolism^(20)^. The reduction in lean body mass, lipolysis and sympathetic nervous system dysregulation causes an alteration in energy balance, which may lead to obesity-related complications^(21)^. Furthermore, several studies have also reported that individuals with SCI consume relatively more dietary fat than recommended^(20,22–24)^. It is worth noting that exercising lower extremity muscles using functional electrical stimulation predominantly relies on carbohydrate consumption over fat as a primary source of energy^(25)^. Encouraged fat utilisation may require low-intensity functional electrical stimulation or neuromuscular electrical stimulation resistance training^(15,24–26)^. The latter has been shown to be effective in reducing ectopic adiposity, primarily intramuscular fat or visceral adiposity.

Furthermore, it is important to note that unchanged dietary habits following SCI without considering the balance between energetic intake and energy expenditure may lead to weight gain and predisposes individuals with SCI to obesity and cardiometabolic risk factors^(13,27,28)^. Therefore, an accurate measure of basal metabolic needs or BMR through indirect calorimetry serves as an important strategy for developing personalised nutritional plans for individuals with SCI and preventing obesity-related comorbidities. Obese individuals with SCI (BMI > 22 kg/m^2^) are predisposed to a wide range of health consequences including but not limited to glucose intolerance, insulin resistance, diabetes mellitus, hypertension, pressure ulcers and CVD^(9,28,29)^. Therefore, it is important to identify strategies to mitigate these adverse effects following SCI. To appropriately address this heightened obesity-related risk after SCI, SCI-specific classifications must be used because relying on AB classification systems may underestimate the prevalence of obesity in persons with SCI. The traditional understanding of obesity was previously defined as an excess accumulation of percent body fat with cut-off values for men greater than 22 % in men and 35 % in women^(9)^. However, since the 1990’s the WHO defined obesity as an increase in BMI calculated by dividing an individual’s weight (kg) by height squared (m^2^)^(30)^. AB individuals with a BMI greater than or equal to 30 kg/m^2^ are considered obese and at risk of cardiovascular diseases, yet BMI is still often applied to persons with SCI, which can severely underestimate obesity. Laughton *et al.*, showed that current BMI cut off values (≥ 30 kg/m^2^) fail to identify obese individuals in the SCI population and clearly provided evidence showing the need to lower BMI cut-off values (≥ 22 kg/m^2^)^(31)^. Gater *et al.* supported this lower BMI cut-off in a sample of 473 veterans that using the WHO standard of BMI (≥ 30 kg/m^2^), 26·9 % of participants were classified as obese, whereas reducing the BMI threshold to 22 kg/m^2^ showed an increase in obesity by 76·7 %^(32)^. It is important to note that the use of BMI as a determinant of obesity for SCI populations does not take into account fat-free mass (FFM), fat mass (FM) or percentage of intramuscular fat. Sumrell *et al.*, developed an SCI-specific waist circumference cut-off value of 86·5 cm for individuals with motor complete SCI. The authors reported that seated/supine circumferences are associated with central adiposity and biomarkers of cardiometabolic disease risk in persons with SCI^(17)^. In addition, quantification of regional abdominal adiposity after SCI using MRI is important considering the cardiometabolic risk factors associated with increasing visceral adiposity. A report by Gorgey *et al.* suggested that a ratio of visceral adipose tissue to subcutaneous adipose tissue greater than 0.4 may increase cardiometabolic risk factors in individuals with SCI^(33)^. Body composition assessment using dual X-ray absorptiometry (DXA) is widely used to assess whole-body and regional composition FFM and FM, and the precision of quantifying total and regional compartments has been previously determined^(34)^. Further, because obesity is so prevalent after SCI, it is necessary to accurately quantify energy expenditure following SCI to determine proper energetic intake and prevent over-feeding^(35)^.

## Indirect calorimetry

**Fig. 1.**
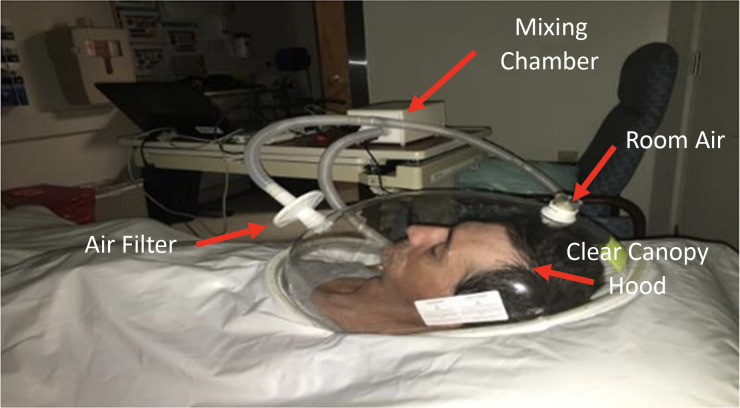
Indirect calorimetry set up for measuring BMR in an individual with complete SCI. The test was administered in a dark thermoneutral environment (22–26°C). The subject is placed under a clear canopy with a plastic drape to eliminate air leakage, which is connected to a COSMED K4B2 mixing chamber.

**Fig. 2.**
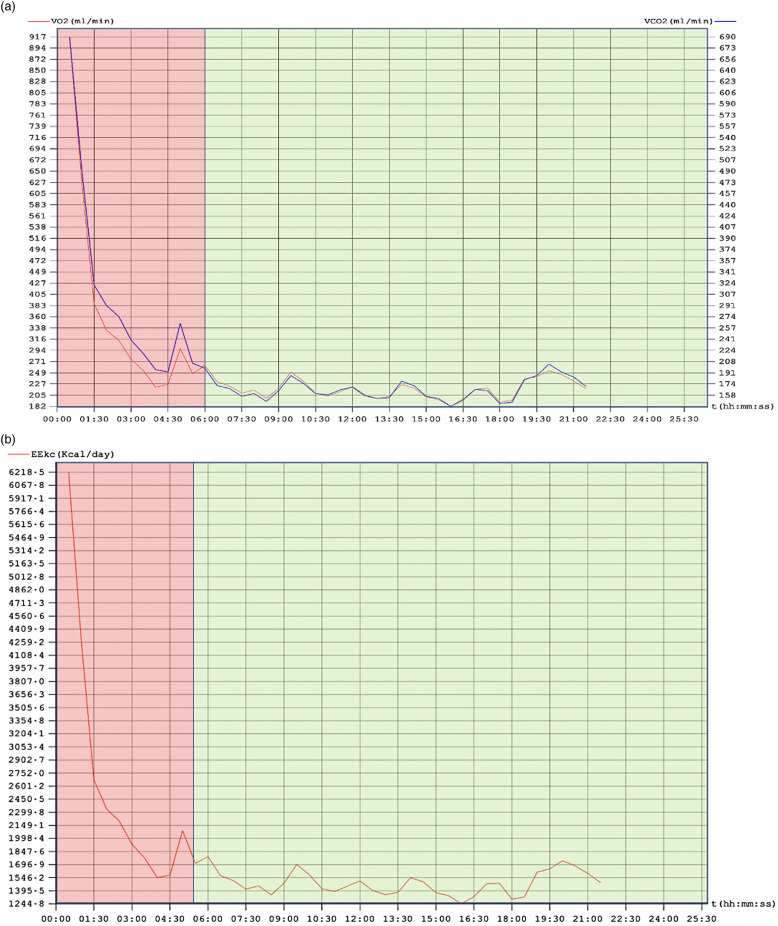
Representative figures displaying ventilatory breath-by-breath measurements and BMR. (a) VCO_2_ and VO_2_ (ml/min) continuous breath-by-breath measurement following an overnight fast (10–12 h) in a dark thermoneutral environment to measure BMR. The first 5 min are discarded (red portion), as determined by the RER ranging from (0·7 to 0·82). (b) BMR (kcal/d) calculated automatically by the COSMED software using the Weir equation (BMR kcal/d) = (VO2 × 3·941) + (VCO2 × 1·11) × 1440). The remaining 15 min (green) are averaged to provide the BMR. Note the oscillation in the measurements suggests that BMR across a 24-hour period may vary in persons with SCI.

Indirect calorimetry is the primary method used to measure metabolic rate from measurements of oxygen consumption and carbon dioxide production. This is, in part, because indirect calorimetry is more practical and feasible due to the use of portable metabolic cart systems, allowing researchers to measure energy expenditure non-invasively in both acute and outpatient settings (See Fig. 1). Regardless of the method used, it is imperative to follow specific guidelines when measuring metabolic rate. For indirect calorimetry, proper calibration of the unit is important to ensure accurate measurements of BMR. In order to achieve an accurate BMR measurement, subjects require an overnight fasted rest for 10–12 h prior to measurement to minimise the contributions of thermic effect of food and physical activity. After the overnight fast, the subject is gently awakened in the early morning (∼06:00 AM) in a dark room with a thermoneutral environment (22–26°C) in order to ensure there is no thermoregulatory effect on heat production. BMR measurement proceeds by placing a clean canopy over the subject’s head while lying in a supine position for approximately 20 min (see Fig. 1). Metabolic cart systems are connected to a mixing chamber that exerts negative pressure to measure the continuous level of inspired and expired gas volumes produced by the participant (see Fig. 2). Acquisition of BMR requires approximately ∼20–25 min, and it is advisable to closely monitor the respiratory exchange ratio (VCO2/VO2 = 0·70–0·82; Fig. 2(a). In contrast, a participant undergoing an RMR measurement does not require an overnight stay as only a short period of rest time is required for RMR measurement (10 to 20 min) prior to RMR acquisition^(36)^. As previously mentioned, BMR and RMR primarily differ in testing protocols, as both are non-invasively measured through indirect calorimetry. In the literature, the terms BMR and RMR are often used interchangeably with basal and resting energy expenditure (BEE and REE). Standardisation of the terminology used in the literature should be considered, as the use of different terminology may lead to confusion.

## BMR *v*. RMR

The imbalance between energy intake and expenditure is important for determining recommended individualised dietary plans that mitigate fluctuations in body weight after SCI. Energy intake can be defined as the total energy content of foods consumed, which also reflects the percentage of macronutrients (carbohydrates, protein, fat and alcohol) consumed daily^(9)^. Recently, the *Clinical Practice Guidelines on Identification and Management of Cardiometabolic Risk after SCI (PVA)* developed recommended nutritional guidelines for individuals following SCI^(37)^. The guidelines promote energetic assessment (BMR or RMR) using indirect calorimetry to accurately measure energy needs in persons with SCI. In addition, the Dietary Guidelines for Americans and the PVA guidelines focus on providing healthy dietary patterns rather than emphasising macronutrient restriction in diets following SCI. To accurately measure daily energy needs, total daily energy expenditure (TDEE) needs to be calculated using the following equation.




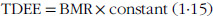




This constant reflects both the thermic effect of food and non-leisure time physical activity^(38)^.

TDEE is the total energy used by an individual during a 24-hour period and is divided into three main components: BMR, thermic effect of food and physical activity energy expenditure. BMR is the largest contributor to TDEE (75–80 % in persons with SCI) and is defined as the minimum daily energy metabolism that individuals require to support essential functions of life, which include breathing, circulation, nutrient processing and cell production^(10,39)^ (see Table 1). Metabolic rate can be measured as BMR or RMR; however, it is important recognise the differences between these indices and understand that these terms are not interchangeable. RMR is defined as the energy required by the body in a resting condition as opposed to a basal condition. Both BMR and RMR are measured following an overnight fasting period of 8–12 h and require abstaining from exercise, caffeine, nicotine and food to remove the effect of thermogenesis carried on by the consumption of food.^(40,41)^. RMR is measured after the participant wakes up and travels to the testing site, undertaking a short resting period prior to the test being conducted. BMR is more precise than RMR due to a more stringent testing protocol and is measured in the morning upon awakening before the participant has performed any movement after an overnight fast for 10–12 h. A study by Wu *et al.* exemplified differences in BMR and RMR in 251 normal weight, overweight and obese AB individuals (BMR: 1429, 1609, 1778 kcal/d (1 kcal = 4.184 kJ) *v*. RMR: 1522, 1712, 1885 kcal/d, respectively)^(42)^. The current evidence clearly shows that BMR is lower in persons with SCI and accounting for this difference is of paramount significance in combating the prevalence of obesity in persons with SCI.

**Table 1.** BMR in spinal cord injury literature

First Author (Year)	Group/Subgroup	LOI	AIS	TSI (years)	sd	*n*	Sex	Age (years)	sd	Height (cm)	sd	Weight (kg)	sd	Methods, Position	Rest time	Medical Device	Measured BMR (kcal/d)	sd
Abel *et al.* (2008)	Tennis (Para) Rugby (Tetra) Basketball (Para)	C5-L3		14·5 10·6 17·0	8·7 5·1 12·6	14 12 10	M M M	35·6 31·7 38·9	5·5 3·3 5·8	181·1 184·8 178·4	5 6·1 12·5	75·4 73·7 73·9	11·4 12·7 20·6	BMR/Supine	30 min. Minimum	Metabolic Cart	BMR: 1603 BMR: 1524 BMR: 1505	307 310 360
Scient *et al.* (1993)	Tetra (Group A) Tetra (Group B) Able-bodied Control	C4-C7	Frankel A	5 4	2 1	6 3 6	M M M	27 28 24	2 3 2	183·0 185·0 188·0	20 20 20	68 66 70	2 2 5	BMR × Position not stated	Basal (Upon awakening)	Metabolic Cart	BMR: 1322 BMR: 1218 BMR: 1630	83 165 41
Bauman *et al.* (2004)	All	C5-L2	Complete/ Incomplete	15	9	13	M/F	37	8·0	174	12	69·9	18	BEE/Supine & REE/Seated	30 min. Minimum	Metabolic Cart	BMR: 1387	268
Chun *et al.* (2017)	All Tetra Para	Tetra/ Para	ASIA A-B ASIA A-B ASIA A-B	12·2 13·4 10·8	7·4 7·6 7·1	50 27 23	M/F M/F M/F	41·9 42·1 41·7	10·7 9·4 12·2					BMR/Supine	20 min	Metabolic Cart	BMR: 1275 BMR: 1313 BMR: 1250	235·2 22·1 31·5
Gorgey *et al.* (2010)	All	C6-T11	ASIA A-B	11	7	10	M/F	33	7	176	11	72	11	BMR/Supine	20–30 min	Metabolic Cart	BMR: 1256	231
Gorgey & Gater (2011)	All Tetra Para	C5-T11 C5-C7 T4-T11	ASIA A-B ASIA A-B ASIA A-B			32 7 25	M M M	36	9	177		74	14	BMR/Supine	20–30 min	Metabolic Cart	BMR: 1431 BMR: 1259 BMR: 1483	345 204 365
Gorgey *et al.* (2015)	All Tetra Para	C5-T10 C5-C7 T3-T10	ASIA A-B ASIA A-B ASIA A-B			16 6 10	M M M	38 39 38	9 8 8	180 181·4 180	7	84 79 87	14 14 14	BMR/Supine	20 min	Metabolic Cart	BMR: 1494 BMR: 1411 BMR: 1526	34 10 34
Gorgey *et al.* (2016)	All Exercise Control	C6-T11 T4-T10 C6-T6	ASIA A-B ASIA A-B ASIA A-B	9·4 13·3 4·7	6·9 9·3 4	11 6 5	M M M	38 40·5 35	7·2 7 7·5	177·5 177 178	5·5 10 10	80·9 85 76	12·8 13 12·5	BMR/Supine	20 min	Metabolic Cart	BMR: 1323 BMR: 1470 BMR: 1147	228 173 403
Gorgey *et al.* (2018)	All Males Females	C6-T11 C7-T8 C6-T11	ASIA A-B ASIA A-B ASIA A-B	10·5 11 10	10·6 10·8 10·5	16 8 8	M/F M F	38·3 37·5 39	11 9 13	171·8 180·5 163	7·5 9 6	81 87 75	14·3 21 17·5	BMR/Supine	Basal (Upon awakening)	Metabolic Cart	BMR: 1394 BMR: 1367 BMR: 1421	450 396 503
Gorgey *et al.* (2019)	TRT + Exercise TRT Only	C5-T11 C6-T11	ASIA A-B ASIA A-B	10 7	9 6	11 11	M M	37 35	13 12	180 180	7 7	80·6 78	15·5 9	BMR/Supine	Basal (Upon awakening)	Metabolic Cart	BMR: 1443 BMR: 1519	231 311
Nightingale & Gorgey (2018)	All Tetra Para	C5-L1	ASIA A-B	35	11	30 9 21	M	35	11	178·0	5	74·5	14·1	BMR/Supine	20–30 min	Metabolic Cart	BMR: 1499 BMR: 1467 BMR: 1497	162 178 148
O’Brien *et al.* (2018)	All	C5-T11	ASIA A-B	8	8	22	M	36	10					BMR/Supine	20–30 min	Metabolic Cart	BMR: 1547	177
Farkas *et al.* (2019)	Tetra Para	C4-C8 T2-L1	ASIA A-B ASIA A-B	13·4 15·5	11·6 11·4	13 28	M/F M/F	45·9 43·4	10·4 11·4	179·9 175·5	8·4 7·5	77·1 85·4	17·6 23·1	BMR/Supine	20 min	Metabolic Cart	BMR: 1224 BMR: 1517	390 398
Cox *et al.* (1985)	All	Tetra/Para		0·18	0·04	22	M/F	29·8				67·9				Metabolic Cart	BMR: 1324	
Yilmaz *et al.* (2007)	AIS A AIS B Tetra Para	Tetra Para	ASIA A ASIA B Complete Complete			22 8 11 19	M M M M	31·9 32·5 28·6 34·0	10·8 10·6 10·4 10·3					BMR/Supine	10 min	Metabolic Cart	BMR: 1433 BMR: 1170 BMR: 1129 BMR: 1499	488 394 300 508

SCI, spinal cord injury; REE, resting energy expenditure; BEE, basal energy expenditure; LOI, level of injury; AIS, American Spinal Injury Association Impairment Scale; TSI, time since injury; Para, paraplegia; Tetra, tetraplegia; P, pressure; NP, no pressure; TRT, testosterone replacement therapy.

Blank spaces indicate data was not provided in the study; data are presented as mean ± standard deviation.

## Differences between BMR and RMR

We comprehensively evaluated the studies that measured BMR^(3,7,10,14,15,38,41–49)^ (*n*=15; Table 1) and RMR^(6,20,24,27,45,50–66)^ (*n*=22; Table 2) to accurately calculate the differences between both metabolic variables. In addition, the average height calculated for studies that measured BMR was 178·9 cm and 165·6 cm for studies that measured RMR. Weight distribution was also noted, as the BMR studies had an average weight of 75·4 kg and 80·5 kg for studies that measured RMR. The mean age for studies that measured BMR was 35·9 years and 40·5 years for studies that measured RMR. In both the studies that measured BMR and RMR, sex distribution was 90 % male and 10 % female. The criteria for classifying the studies into either BMR or RMR relied primarily on analysing the described methods to determine whether the SCI participants had BMR measured immediately upon awakening prior to any movement or an RMR measurement was performed after the subject wakes up and travelled to the testing site, and then undertakes a short resting period prior to assessment. We are aware that some of the reported studies referred to BMR as RMR; these studies based on the described methods accurately measured BMR^(3,47)^.

**Table 2.** RMR in spinal cord injury literature

First Author (Year)	Group/Subgroup	LOI	AIS	TSI (years)	sd	*n*	Sex	Age (years)	sd	Height (cm)	sd	Weight (kg)	sd	Methods, Position	Rest time	Medical Device	Measured RMR (kcal/d)	sd
Alexander *et al.* (1995)	Pressure Sore (Para) No Pressure Sore Para Control		Complete/ Incomplete			14 24 23	M M M	53 50 54	3 3 3	182·0 180·0 172·0	1 1 1	74·0 84·6 79·0	4·2 3·6 3·0	RMR/Supine & Seated	30 min. Minimum	Metabolic Cart	RMR: 1891 RMR: 1780 RMR: 1751	97 62 55
Aquilani *et al.* (2001)	Para Control		ASIA A			10 5	M M/F	42·1 27·6	18·7 7·7			64·8 54·3	11·3 4·9	RMR × Position not stated	not stated*	Metabolic Cart	RMR: 1469 RMR: 1059	217 46
Bauman *et al.* (2004)	All	C5-L2	Complete/ Incomplete	15	9	13	M/F	37	8·0	174	12	69·9	18	BEE/Supine & REE/Seated	30 min. Minimum	Metabolic Cart	RMR: 1682	388
Bauman *et al.* (2011)	TRT Control	Tetra/ Para	ASIA A-C ASIA A-C	13 12	10 9	11 11	M M	43 35	6·0 9·0	179 174	7 4	82·9 79·6	12·6 9·2	REE/Supine	20 min	Metabolic Cart	RMR: 1328 RMR: 1319	262 112
Bucholz *et al.* (2003)	Male (Para) Female (Para) Complete Incomplete	Para Para Para Para	Complete/ Incomplete Complete Incomplete	10·4 16·1	8·1 11·1	17 10 17 10	M F M/F M/F	38·7 31·7 35·5 37·1	10·7 6·0 10 9·7	173 154 164 170	7·1 10·6 13·4 9·9	71·2 57·5 65·5 67·3	14·6 14·2 15·2 17·3	RMR/Supine	not stated*	Metabolic Cart	RMR: 1555 RMR: 1245 RMR: 1417 RMR: 1480	165 176 214 249
Bucholz *et al.* (2003)	Para Able-bodied Control	Para	Complete/ Incomplete	11	10	28 34	M/F	33·9 29·1	9·2 7·6	164·5 173·0	13·0 10·2	65·5 70·7	16·3 10·6	RMR/Supine	not stated*	Metabolic Cart	RMR: 1472 RMR: 1677	228 223
Broad *et al.* (2020)	All		Complete/ Incomplete			14	M	31·0	6·0			66·43	10·45	REE/Supine	30 min	Metabolic Cart	RMR: 1735	257
Collins *et al.* (2010)	All Tetra Para	C5-L4 C5-C8 T1-L4	ASIA A-D ASIA A-D ASIA A-D	13·2 10·5 15·8	12·9 11·5 14·3	66 32 34	M M M	52·3 53·0 51·6	8·4 14·3 12·3	178·0 178·7 177·3	7·0 6·8 7·1	76·5 78·2 74·9	17·8 18 17·6	RER/Supine	not stated*	Metabolic Cart	RMR: 1422 RMR: 1411 RMR: 1433	273 315 233
Tanhoffer *et al.* (2012)	All	C4-T12	ASIA A-C	10	8	14	M/F	40	13	181		79	15	BMR/Supine	30 min	Metabolic Cart	RMR: 1433	228
Fellieter *et al.* (2017)	All Tetra Para	C4-T12	ASIA A-C			8 16	M/F	32·0 42·1	12·3 14·3	175·6 180·3	7·2 5·3	73·1 76·6	4·9 3·4	REE/Supine	15 min	Metabolic Cart	RMR: 1582	241
Gorgey *et al.* (2012)	All Exercise + Diet Diet	C5-T11 C5-T10 T4-T11	ASIA A-B ASIA A-B ASIA A-B	12·4 16 8	9·4 9 10	9 5 4	M M M	35 36 33	9 9 10			74·9 74 76	11·3 14 8	RMR/Supine	20–30 min	Metabolic Cart	RMR: 1554 RMR: 1363 RMR: 1793	250 132 397
Hayes *et al.* (2002)	SCI Able-bodied control	Tetra/ Para				7 7	M/F M/F	35·9 36·0	7·9 6·8	175		68·5 68·4	23·1 25·2	REE/Supine	not stated*	Metabolic Cart	RMR: 1390 RMR: 1491	245 346
Holmlund *et al.* (2018)	Tetra Para	C5-C8 T7-T12	ASIA A-B	15·3 15·8	10·9 11·3	26 38	M/F M/F	41·5 43·0	14 11·5	178 177	0·09 0·10	65·3 73·7	12·9 15·1	REE/Supine	not stated*	Metabolic Cart	RMR: 1132 RMR: 1218	217 244
Jeon *et al.* (2003)	SCI Able-bodied control	C5-C7				7 7	M M	38·3 38·0	3·1 4·4	178 176·4	8·6 3·1	86·5 91·7	7 4·3	RMR/Supine	30 min	Metabolic Cart	RMR: 1451 RMR: 1848	241 258
Lee *et al.* (1985)	All (Para) Hypometabolic Normometabolic Hypermetabolic	T4-L2	Complete/ Incomplete	17·8 15·6 19·3 18·6	12·3 9·3 14·3 12·0	17 6 5 6	M M M M	42·8 44·2 41·0 48·3	12·7 14·6 5·2 15·5	177·8 178·0 175·3 180·3	6·0 8·9 4·5 5·9	73·9 81·4 77·1 63·7	15·7 12·7 18·5 8·9	REE × Position not stated	not stated*	Metabolic Cart	RMR: 1602 RMR: 1588 RMR: 1757 RMR: 1786	232 209 283 255
Liu *et al.* (1996)	P Ulcer (Tetra) NP Ulcer (Tetra) Control (Able-bodied)			10 15	2 3	16 16 16	M M M	40 40 43	3 2 3	178 177 173	2 1 2	80·1 75·6 82·9	6·5 3·7 4·3	REE/Seated	30 min	Metabolic Cart	RMR: 1775 RMR: 1538 RMR: 1847	74 66 67
Monroe *et al.* (1998)	Control (Able-bodied) SCI	C6-L3	Frankel A	9	2	59 10	M M	31·9 35·5	7·1 8·0	176·8 179·7	6·9 5·4	89·9 70·1	23·6 17·2	RMR/Supine	not stated*	Metabolic Cart	RMR: 2211 RMR: 1756	317 64
Nightingale *et al.* (2017)	All	T1-L4	ASIA A-B	15	10	33	M/F	44	9			76·1	12·5	RMR/Supine	20 min	Douglas Bag	RMR: 1481	32
Pelly *et al.* (2017)	Para Able-Bodied Control	T3-L5	Complete/ Incomplete			7 7	M M	31·3 32·7	7·3 7·2	173·1 179·4	18·5 5·4	72·0 76·1	15·2 8·5	REE/Supine	10–20 min	Metabolic Cart	RMR: 1538 RMR: 1664	139 34
Perret & Stoffel-Kurt (2011)	Acute Chronic	C5-T12	ASIA A-B	0·44 4·6	0·20 1·8	12 12	M/F M/F	27·7 28·8	7·1 6·5	178·3 174·7	7·9 6·1	69·8 66·4	12·2 14·5	REE/Supine	5–10 min	Metabolic Cart	RMR: 1414 RMR: 1304	327 232
Sedlock and Laventure (1990)	All	T4-L1		7·4	3·3	4	M	27·7	2·3	180·5	2·5	73·3	4·9	RMR/Supine	60 min	Metabolic Cart	RMR: 1530	330
Spungen *et al.* (1993)	All	Para		10	2	12	M	42	3	179	2	87·6	4·2	RMR/Seated	30 min	Metabolic Cart	RMR: 1854	70

SCI, spinal cord injury; REE, resting energy expenditure; BEE, basal energy expenditure; LOI, level of injury; AIS, American Spinal Injury Association Impairment Scale; TSI, time since injury; Para, paraplegia; Tetra, tetraplegia; P, pressure; NP, no pressure; TRT, testosterone replacement therapy.

Blank spaces indicate data was not provided in the study; data are presented as mean ± standard deviation.

The results showed that BMR was equivalent to 1397 ± 139 kcal/d, whereas RMR is equivalent to 1527 ± 188 kcal/d. This means that BMR is 9 % lower than RMR with an absolute difference of 130 kcal/d (the difference between 1527 and 1397 kcal/d). This difference can be translated to 3900 kcal/month or 46 800 kcal/year. Therefore, this overestimation by relying on RMR may lead to a 6·1 kg (0·5 kg = 3500 kcal) increase in body weight.

The reduction in muscle and LM after SCI has been well established in comparison to the non-injured population^(18)^. However, the dramatic changes in muscle mass depend on several factors, including the level of injury and severity of the lesion, among others^(67)^. For instance, Bauman *et al.* showed differences in BMR and RMR in thirteen pairs of monozygotic twins with and without SCI and reported higher RMR values (SCI twin: 1682 kcal/d *v*. non-SCI twin: 1854 kcal/d) in comparison to BMR values (SCI twin: 1387 kcal/d *v*. non-SCI twin: 1660 kcal/d)^(45)^. Importantly, the authors demonstrated that RMR was 20 % higher than BMR in SCI twins, while RMR was found to be 11 % higher than the measured BMR for non-SCI twins. Both BMR and RMR were significantly lower in the twins with SCI compared with non-SCI twins, more specifically BMR was 18 % lower in SCI twins compared with non-SCI twins, while RMR was 10 % lower for SCI-twins. Importantly, when BMR and RMR were adjusted to FFM, BMR/FFM was not different between groups (SCI twin: 30·4 kcal/kg *v*. non-SCI twin: 29·9 kcal/kg), while RMR/FFM was 3·5 kcal/kg higher for SCI twins compared with non-SCI twins (SCI twin: 36·9 kcal/kg *v*. non-SCI twin: 33·4 kcal/kg). Likewise, when BMR and RMR were adjusted to body mass, (BMR: SCI twin: 20·4 kcal/kg *v*. non-SCI twin: 21·5 kcal/kg) and (RMR: SCI twin: 24·9 *v*. non-SCI twin: 24·2 kcal/kg) were not significantly different between both groups. Additionally, the authors found significant linear relationships between FFM with BMR and RMR for both SCI twins and non-SCI twins^(45)^. Bauman and colleagues clearly demonstrated that following chronic paralysis, greater reductions in LM is directly related to the reduction in energy expenditure following SCI, and importantly the authors measured both BMR and RMR in the same sample of individuals, whereas all other studies evaluated either measured BMR or RMR^(45)^. Similarly, Wu *et al.* examined differences in BMR and RMR in AB individuals and showed that in normal-weight AB individuals RMR was 7 % higher than BMR (112 kcal/d difference), which is slightly lower than the 11 % difference observed by Bauman *et al.* with the non-SCI group^(42,45)^. In addition, RMR was 5 % higher for overweight individuals and 6 % higher than BMR for the obese group^(42)^. The authors also noted that the absolute BMR and RMR was significantly different for the three groups (normal weight, overweight, obese), but after adjusting to FFM no significant differences were found^(42)^.

In addition, there is a notable difference between the absolute metabolic rate in paraplegic compared with tetraplegic individuals. Gorgey *et al.* reported a difference of 224 kcal/d in BMR between tetraplegic and paraplegic participants; however, after adjustment to FFM, BMR was not different between both groups (29·4 *v*. 29·4 kcal/kg, respectively)^(41)^. A study by Yilmaz *et al.* also reported a significant difference of 370 kcal/d in BMR between tetraplegic and paraplegic participants, and BMR/LM was modestly 2·5 kcal/kg lower for the tetraplegic group (33·9 *v*. 36·5 kcal/kg, respectively)^(68)^. Farkas *et al.* reported a significant difference in BMR between tetraplegic and paraplegic participants (1224: *v*. 1517: kcal/d, respectively), BMR/LM was not different between groups (29·9 *v*. 30·3 kcal/kg, respectively)^(38)^. In addition, Gorgey *et al.* reported a non-significant difference in BMR between tetraplegic and paraplegic participants (1411: *v*. 1526: kcal/d, respectively), and BMR/LM was 3·0 kcal/kg higher for the tetraplegic group^(10)^. However, a study by Collins *et al.* did not report any significant differences in RMR between tetraplegic and paraplegic participants (1411: *v*. 1433: kcal/d, respectively)^(56)^. In addition, the authors did not measure LM or FFM, however when RMR was divided by body mass (kg), RMR/body mass was 1·1 kcal/kg lower in the tetraplegic group compared with the paraplegic group (18·0 *v*. 19·1 kcal/kg, respectively). Similarly, Chun *et al.* examined only motor complete SCI, and although a non-significant 63 kcal/d difference in BMR was demonstrated between tetraplegic and paraplegic participants (1313 *v*. 1250 kcal/d), BMR/FFM was 1·7 kcal/kg lower for the tetraplegic group compared with the paraplegic group (29·7 *v*. 31·4 kcal/kg, respectively)^(46)^. Discrepancies in energy expenditure between these studies may be attributed to methodological differences, heterogeneity between samples (e.g., incomplete *v*. complete SCI), sample size and/or population demographics. For instance, Chun *et al.*, Gorgey and Gater, Farkas *et al.* and Yilmaz *et al.* examined chronic motor complete SCI and measured BMR, whereas Collins *et al.* included both complete and incomplete SCI participants in their samples and measured RMR as opposed to BMR. The current findings may suggest that the metabolic rate is similar between paraplegic and tetraplegic groups after adjusting to total body mass, FFM and LM. However, it is worth noting that although the range in adjusted BMR to FFM or LM was narrow in some studies (0·0 to 0·5 kcal/kg of FFM or LM)^(3,38)^, while other studies demonstrated a wider range from 2·5 to 9·0 kcal/kg of FFM between tetraplegic and paraplegic groups^(10,46,68)^. As previously mentioned, differences in reported metabolic rate may be attributed to methodological differences, heterogeneity of the studied samples, sample size and population demographics/ethnicity.

Moreover, based on the available literature, we evaluated differences between both BMR and RMR between persons with paraplegia and tetraplegia. Six studies (6/24) evaluated BMR by level of injury, and persons with paraplegia showed an average BMR of 1462 ± 105 kcal/d, whereas tetraplegia had an average BMR of 1301 ± 124 kcal/d^(3,7,10,38,46,68)^. This 162 kcal/d (∼12 %) difference can be simply explained as lower LM in persons with tetraplegia compared with paraplegia^(5)^. Likewise, the two studies that evaluated RMR by level of injury showed that paraplegic individuals had an average RMR of 1326 ± 152 kcal/d, whereas tetraplegia had an average RMR of 1272 ± 197 kcal/d^(56,59)^. The reported differences in RMR between individuals with paraplegia and tetraplegia can also be attributed to the fact that individuals with motor-complete tetraplegia experience a greater loss of LM. Singh *et al.* reported a 6 % lower LM in the trunk and ∼10 % lower LM in the arms for individuals with tetraplegia^(67)^. Similarly, Rankin *et al.* reported that individuals with tetraplegia have a 13 % smaller trunk muscle cross-sectional area compared with those with paraplegia, with DXA trunk-LM predicting 37 % of the variance in BMR^(69)^. Such reductions in LM in individuals with tetraplegia are important to consider when organising dietary plans for individuals with SCI, as less metabolically active tissue can influence either BMR or RMR.

The differences in measured metabolic rate between tetraplegic and paraplegic individuals warrant the need for more research. Previous reports have shown that BMR is approximately ∼20 % lower compared with RMR in persons with SCI, while only ∼11 % lower than RMR in persons without SCI^(41,45)^. Based on the current findings, BMR was found to be 9 % lower compared with RMR in persons with SCI. The discrepancy may be stemmed from classifying the reported studies into either BMR or RMR. Importantly, since RMR has been shown to be 20 % higher than BMR in SCI, using RMR as a predictor of energy expenditure instead of BMR in persons with SCI may overestimate dietary needs by roughly 400 kcal/d assuming a 2000 kcal/d diet, which could lead to inappropriate nutrient intake recommendations that may exacerbate the risk for obesity and secondary cardiometabolic complications^(41,45)^. Moreover, previous reports have indicated that many individuals with SCI have a disproportionate energetic intake relative to their energy requirements, which consequently results in positive energy balance that may increase the risk of obesity in individuals with SCI^(22,32)^. Several studies have shown that individuals with chronic SCI require a 10 % lower energetic intake compared with AB individuals, which equals 200 kcal/d assuming a 2000 kcal/d diet^(45,53,63)^. Therefore, an overestimation of energetic intake using RMR may lead to obesity, considering that previous reports have shown that an additional 100–200 kcal/d should be restricted to promote negative energy balance^(11)^.

While BMR is more precise than RMR, it is important to emphasise that RMR is often more practical and feasible to measure due to its less stringent protocol, especially with the advent of portable metabolic carts. Therefore, BMR measurement is ideal, but RMR is an acceptable proxy if appropriately conducted and standardised. Fellieter *et al.* investigated the changes in RMR and body composition over time in individuals admitted for acute treatment after SCI and showed that the average RMR from 2 weeks of admission to 130 weeks decreased significantly (1582 *v*. 1291 kcal/d, respectively)^(27)^. Importantly, RMR was measured instead of BMR, possibly because RMR is measured under less strict conditions, as individuals in the acute phase spent more time in the intensive care unit. Several participants in the study were unable to undergo RMR assessment due to spinal shock, as they experienced symptoms of hypotension that required clinical intervention through the delivery of high fluid volumes and vasopressors to mitigate hypotension^(27)^. Likewise, under certain conditions, it may not be feasible to measure metabolic rate, in which case population-specific prediction equations may be used to estimate BMR.

## Population specific prediction equations

The use of predictive equations as a surrogate for metabolic rate measurement allows clinicians to estimate the energy needs for individuals with SCI. Prediction equations rely on assumptions and have many limitations, and the most widely used prediction equations are not reliable in SCI, given that they are derived from uninjured populations. Prediction equations derived from populations without SCI include the following: Harris Benedict, 1919; Schofield, 1985; and Mifflin-St., 1990^(70–72)^. A review by Nevin *et al.* reported that prediction equations derived from AB individuals overestimate measured metabolic rate from 4 % to 92 % in individuals with SCI^(73)^. Cox and colleagues showed that using the Spanier and Shizgal equation which predicted RMR by multiplying body mass (kg) by 45 kcal/d resulted in a predicted RMR of 3056 kcal/d, resulting in an overprediction of RMR by 92 % compared with the measured RMR of 1589 kcal/d^(73–75)^. These equations typically incorporate age, weight and height, which are primarily used to estimate BMR in AB individuals. The predicted values of RMR and BMR reported by Bauman and colleagues were found to be significantly higher than the measured metabolic rate by indirect calorimetry^(45,52)^. A recent systematic review by Farkas *et al.* evaluated the accuracy of predicted metabolic rates in comparison to measured metabolic rates through indirect calorimetry. The authors confirmed that several prediction equations derived from AB individuals overestimate metabolic rate and energetic requirements for individuals with SCI^(1)^. Importantly, the previously described predictive equations do not factor in LM, FM or regional adipose tissue.

Recent SCI population-specific equations developed by Chun *et al.* & Nightingale & Gorgey, more accurately estimate BMR by incorporating measures of FFM via DXA^(7,46)^. Additionally, further incorporation of anthropometric measurements such as weight, height and circumferential methods have been shown to improve estimations of BMR by 8 % (r^2^ = 0·77)^(7)^. Similarly, Chun *et al.* reported a mean difference of 5·4 kcal/d between the measured and predicted metabolic rate after accounting for FFM^(46)^. These two SCI-specific prediction equations offer the best estimate of BMR in adults with chronic SCI; however, difficulty obtaining FFM using DXA may limit the feasibility predicting BMR using these methods. Gorgey *et al.* suggested that a simple measurement of body weight (kg) can be used to predict whole-body FFM in men with chronic complete SCI using the following prediction equation: whole-body FFM = 0·288 × body weight (kg) + 26·3^(76)^. Chun *et al.*, found no statistical differences between estimated and DXA-measured values of FFM in thirty-eight male participants using the previously described prediction equation^(46,76)^. In the absence of detailed body composition information, utilisation of anthropometric measurements (height, weight and transverse abdominal diameter) offers a useful method in predicting BMR but yields a lower R^2^ value of (r^2^ = 0·57)^(7)^. Furthermore, prediction equations may be used to estimate TDEE from measured BMR or RMR.

Recently, Farkas *et al.* performed detailed calculations using a metabolic equivalent of 2·7 ml of oxygen/kg of body weight/min as opposed to 3·5 ml/kg per min that is commonly used in the general population^(38)^. The authors developed a novel SCI-specific correction factor of 1·15 instead of 1·2 to estimate TDEE from BMR using one metabolic equivalent of 2·7 ml of oxygen/kg of body weight/min^(38)^. An accurate estimate of TDEE should be considered after measuring or estimating BMR, which further allows clinicians and dieticians to devise appropriate dietary and exercise regimens that address the individual patient’s needs while combatting obesity and other SCI-associated secondary comorbidities.

Regardless of the mode of measurement, individuals with SCI experience a reduction in metabolic rate, which contributes to obesity and increases risk for several cardiometabolic diseases^(6)^. Therefore, an accurate determination of energy expenditure is important to ensure appropriate recommendations of dietary intake to mitigate this heightened risk in persons with chronic SCI. Importantly, different methods of calorimetry (direct *v*. indirect) are commonly used to measure metabolic rates.

## Discussion

The majority of the SCI literature detailed in this review indicates a shift towards measuring RMR, instead of BMR, with the latter being more precise in quantifying energy expenditure (Tables 1 and 2). Metabolic rates are consistently lower in persons with SCI compared with the general population. The vast majority of inconsistencies between BMR/RMR measurements in SCI *v*. AB individuals is attributed to reduced lean body mass following SCI. More specifically, reductions in metabolically active muscle and bone tissue below the level of injury account for significant reductions in energy expenditure and increases in fat tissue. Moreover, several reports have indicated a lower metabolic rate in tetraplegic SCI compared with paraplegic SCI, which has been confirmed in the current review. Since RMR is not measured in a basal state, RMR is generally higher in comparison to BMR for individuals with or without SCI^(41)^. We showed that BMR is 9 % lower compared with RMR in persons with SCI. Across the SCI literature, the mean BMR measured for individuals with SCI was 1397 kcal/d (range:1124 kcal/d–1603 kcal/d), whereas the mean measured RMR was 1527 kcal/d (range:1132 kcal/d–1891 kcal/d) (Tables 1 and 2). Importantly, numerous factors affect metabolic rates in persons with SCI, including type of mobility device (e.g., manual wheelchair, walker, power wheelchair, etc.). For instance, Gorgey *et al.* reported a 15 % difference in BMR comparing manual and power wheelchair users (1551 *v*. 1340 kcal/d, respectively)^(10)^. Although differences in BMR were not statistically significant, possibly due to a limited sample size (*n*=13), these findings do however suggest that manual wheelchair users have a higher level of activity due to the physical exertion of using a manual wheelchair compared with power wheelchair users. Therefore, the type of mobility device should be clearly indicated when reporting metabolic rates in this population.

Additionally, several interventions have shown that increases in lean body mass have resulted in increased metabolic rate in SCI. Bauman *et al.* demonstrated that hypogonadal male SCI participants on testosterone replacement therapy (TRT) (5–10 mg/d) had a significant increase in RMR (1328 *v*. 1440 kcal/d, respectively^(52)^. Likewise, Welle *et al.* reported a 10 % increase in BMR following 3 months of TRT in AB men with muscular dystrophy and a 7 % increase in healthy AB individuals^(77)^. Given these findings, TRT can increase lean body mass, and also significantly improve metabolic rate. Gorgey *et al.* examined the effect of 16 weeks of neuromuscular electrical stimulation resistance training (RT) in combination with low dose TRT (2–6 mg/d) *v*. TRT only and reported a 14–17 % increase BMR following the combination with no change in the TRT-only group of men with complete SCI (1693 *v*. 1502 kcal/d, respectively)^(15)^. The increase in RMR reported by Bauman *et al.* may be due to a higher dose of testosterone administered to participants (5–10 mg/d), whereas Gorgey *et al.* reported no significant changes in BMR in the TRT-only group (2–6 mg/d).

In another study, Gorgey *et al.* showed that following 16 weeks (5 d per week) of either functional electrical stimulation cycling (*n*=3) or arm cycling ergometry (*n*=3), BMR was significantly reduced by ∼17 % following both interventions^(48)^. Nevertheless, the conflicting change in metabolic rate noted in previous studies suggests that changes in BMR are dependent upon the mode of exercise and dietary restriction-induced reductions in metabolic rate in persons with SCI.

Reductions in energetic intake in combination with exercise and/or pharmacological interventions for individuals with SCI have been known to reduce FM and concomitantly increase LM. A recent case report noted a 25 % reduction in energetic intake (440 kcal/d, respectively) in an individual with motor complete T5 SCI in combination with 16 weeks of TRT administration resulting in total body weight reduction by 8 % and total body fat reduction by 29 %^(78)^. These findings demonstrate that accurately determining BMR allows researchers to better understand the role of exercise and clinical interventions in promoting improvements in body composition and health outcomes to combat obesity-related complications in persons with SCI.

The literature reviewed measured BMR and/or RMR non-invasively with indirect calorimetry using a metabolic cart (Tables 1 and 2). As previously stated, BMR is precise, but RMR is often more feasible. Moreover, while direct calorimetry is the gold standard for measuring TDEE, indirect calorimetry is considered the gold standard to measure BMR/RMR to estimate TDEE. In the clinical environment, access to metabolic carts for indirect calorimetry may be limited, and thus the measurement of metabolic rate may not be feasible. The use of predictive equations as a surrogate for indirect calorimetry allows clinicians to estimate energy needs for individuals with SCI; however, commonly used equations do not factor in LM, FM or regional adipose tissue. As previously mentioned, recent SCI population-specific equations have been developed that more accurately estimate BMR through measures of FFM via DXA and anthropometric circumferential measurements^(7)^. However, limited access to advanced imaging techniques (i.e., DXA) may limit the feasibility predicting BMR using this method. Therefore, utilisation of anthropometric measurements (height, weight and transverse abdominal diameter) may offer a useful and feasible method for predicting BMR. Moreover, important work has been done to more accurately estimate TDEE from BMR measurements in persons with SCI^(38)^. This is an important step that further allows clinicians and dieticians to devise individualised dietary and exercise plans to mitigate obesity and SCI-associated secondary comorbidities.

Several methodological differences have been noted while reviewing SCI literature on metabolic rate. The majority of studies measured metabolic rate in a supine position; however, several studies measured metabolic rate in a seated position (Tables 1 and 2). Previous reports have indicated that certain postures require increased muscle tone and may potentially influence the measurement of metabolic rate^(36)^. In contrast, other studies have shown that acquisition of metabolic rate through indirect calorimetry is not substantially influenced by position^(79)^. It is also worth noting that spasticity occurs in more than 80 % of individuals with SCI, which is characterised by involuntary and uncontrolled muscle contractions^(80)^. During the assessment of either BMR or RMR, occurrences of spastic hypertonia may result in increased energy expenditure due to excessive muscle contraction, which may warrant multiple measurements of BMR or RMR using indirect calorimetry for individuals with severe spasticity^(81)^. In majority of the literature reviewed, resting time prior to RMR acquisition was 20–30 min; however, several studies reported resting times ranging from 5 to 20 min. Nevertheless, standardisation and consistency would dramatically improve our understanding of metabolic rate following SCI. Moreover, differences in terminology need to be addressed while describing metabolic rate in persons with SCI, as we have clearly noted that BMR is a more accurate measure in comparison to RMR and these terms are not interchangeable.

Future research should take into consideration the overestimation of energy expenditure when using RMR and use the more precise assessment of BMR instead. If BMR is not feasible, then the dietary recommendations need to properly account for this overestimation. Additionally, it is highly recommended that researchers and clinicians acknowledge the accurate measure of BMR when using SCI-specific prediction equations to predict TDEE, as using RMR may potentially overestimate total energy expenditure. Future studies with larger sample sizes are needed to evaluate the influence of level of injury, completeness of injury and sex on BMR, RMR and TDEE in persons with SCI.
